# Functional and Structural Changes in the Membrane-Bound O-Acyltransferase Family Member 7 (MBOAT7) Protein: The Pathomechanism of a Novel *MBOAT7* Variant in Patients With Intellectual Disability

**DOI:** 10.3389/fneur.2022.836954

**Published:** 2022-04-18

**Authors:** Jiwon Lee, Amen Shamim, Jongho Park, Ja-Hyun Jang, Ji Hye Kim, Jeong-Yi Kwon, Jong-Won Kim, Kyeong Kyu Kim, Jeehun Lee

**Affiliations:** ^1^Department of Pediatrics, Samsung Medical Center, Sungkyunkwan University School of Medicine, Seoul, South Korea; ^2^Department of Computer Science, University of Agriculture, Faisalabad, Pakistan; ^3^Department of Precision Medicine, Graduate School of Basic Medical Sciences, Sungkyunkwan University School of Medicine, Suwon, South Korea; ^4^Department of Laboratory Medicine and Genetics, Samsung Medical Center, Sungkyunkwan University School of Medicine, Seoul, South Korea; ^5^Department of Radiology, Samsung Medical Center, Sungkyunkwan University School of Medicine, Seoul, South Korea; ^6^Department of Physical and Rehabilitation Medicine, Samsung Medical Center, Sungkyunkwan University School of Medicine, Seoul, South Korea

**Keywords:** intellectual disability, *MBOAT7*, autism spectrum disorder, globus pallidus, cerebellar dentate nucleus, molecular modeling

## Abstract

The membrane-bound O-acyltransferase domain-containing 7 *(MBOAT7)* gene is associated with intellectual disability, early onset seizures, and autism spectrum disorders. This study aimed to determine the pathogenetic mechanism of the *MBOAT7* missense variant via molecular modeling. Three patients from a consanguineous family were found to have a homozygous c.757G>A (p.Glu253Lys) variant of *MBOAT7*. The patients showed prominent dysfunction in gait, swallowing, vocalization, and fine motor function and had intellectual disabilities. Brain magnetic resonance imaging showed signal changes in the bilateral globus pallidi and cerebellar dentate nucleus, which differed with age. In the molecular model of human MBOAT7, Glu253 in the wild-type protein is located close to the backbone carbonyl oxygens in the loop near the helix, suggesting that the ionic interaction could contribute to the conformational stability of the funnel. Molecular modeling showed that Lys253 in the mutant protein was expected to alter the surface charge distribution, thereby potentially affecting substrate specificity. Changes in conformational stability and substrate specificity through varied ionic interactions are the suggested pathophysiological mechanisms of the *MBOAT7* variant found in patients with intellectual disabilities.

## Introduction

Membrane-bound O-acyltransferase domain-containing 7 (*MBOAT7*) is also known as lysophospholipid acyltransferase 7, leukocyte receptor cluster gene 4 (*LENG4*), or breast and bladder cancer overexpressed gene 1 (*BB1*). *MBOAT7* was recently reported to be associated with intellectual disabilities and autism spectrum disorders (1). This gene encodes lysophosphatidylinositol acyltransferase 1 (LPIAT1). After the first report of *MBOAT7* variants in 2016, 44 patients were subsequently identified (1–7). These patients showed various phenotypes, such as moderate-to-severe intellectual disability, seizures, and autism spectrum disorder. Various brain imaging abnormalities have been reported, including brain atrophy, polymicrogyria, cerebellar dysgenesis, or T2 hyperintensity of the dentate nuclei, and globus pallidi (1–7) (Supplementary Material 1).

MBOAT7 (LPIAT1) is the predominant enzymes that catalyze the incorporation of arachidonic acid into lysophosphatidylinositol and is involved in phosphatidylinositol acyl-chain remodeling in the Lands cycle (8). MBOAT7 deficiency in this process changes the hepatic lipid composition to increase saturated lysophosphatidylinositol levels, resulting in fatty liver diseases (9). Although it is not evident how MBOAT7 deficiency occurs in the nervous system, it plays a crucial role in murine brain development (1, 10). A previous report showed that LPIAT1 deficiency causes disordered neuronal processes in the cortex and reduced neurite outgrowth *in vitro*. Functional studies using LPIAT1-knockout mice have shown an atrophic cerebral cortex and hippocampus, abnormal cortical lamination, and an increased number of apoptotic cells in the cortex. Arachidonic acid-containing phosphatidylinositol is expected to play an important role in normal cortical lamination during brain development (10). It also affects neurometabolism in the human brain, which may lead to neuronal degeneration and increased gliosis (2).

Although there have been many clinical reports on the various phenotypes in patients with *MBOAT7* variants, the pathophysiological mechanism of the *MBOAT7* variant has not been fully reported. This study aimed to determine, via molecular modeling of wild-type and variant proteins, how the variant affects enzyme activity and causes symptoms in patients.

## Materials and Methods

### Patients

**Figure 1:**
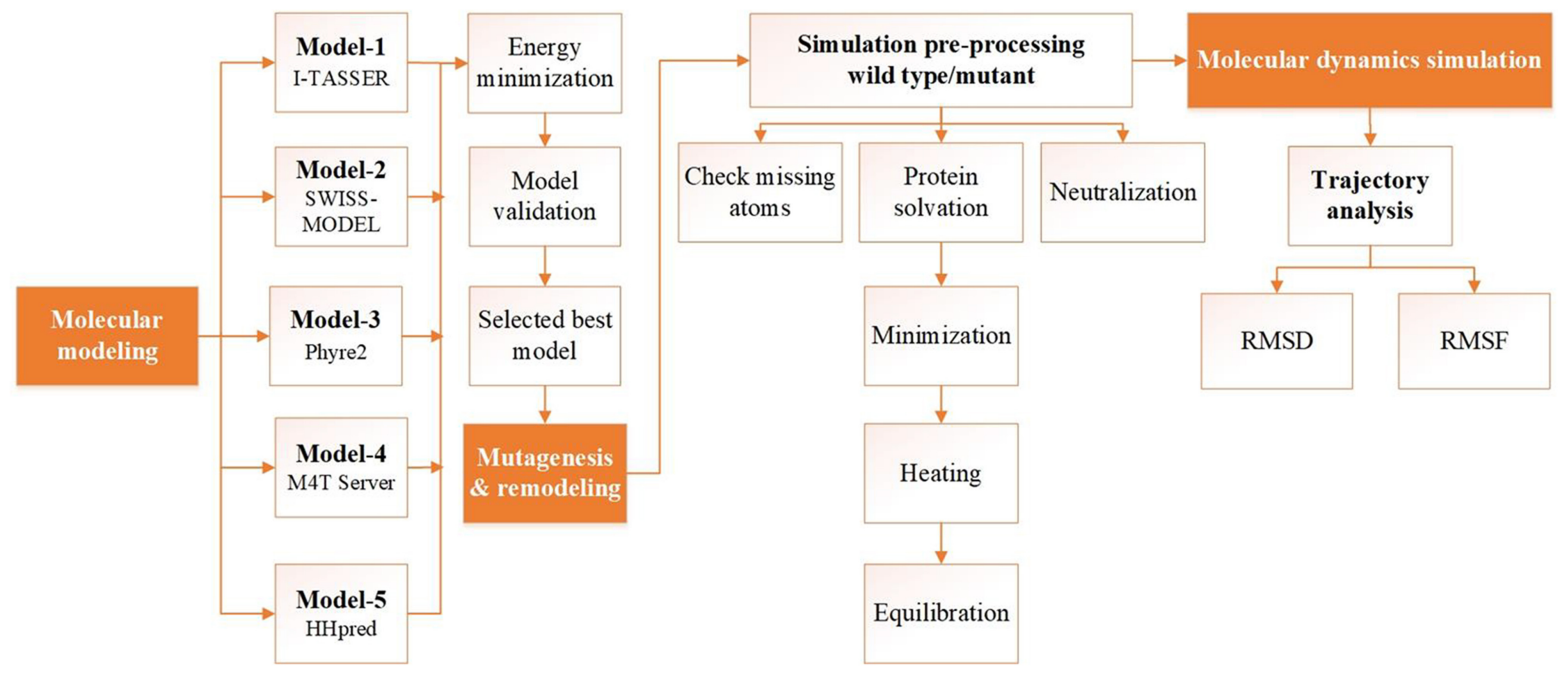
Flowchart of the structure prediction and analysis pipeline. Schematic illustrating the structure prediction and analysis pipeline consisting of model building, validation, and molecular dynamics simulation analysis. Each step corresponding to the protocol is described in the main text.

Three affected patients, three healthy siblings, and their parents, who all came from a consanguineous family from the UAE were included in this study (Figure 1). The parents are distant relatives.

### Clinical Evaluation of the Subjects

The three affected individuals were clinically evaluated by pediatric neurologists and rehabilitation doctors at the Samsung Medical Center, Seoul, Korea. Clinical examinations, routine laboratory testing, electroencephalography (EEG), and brain magnetic resonance imaging (MRI) were performed.

### Whole Exome Sequencing, Whole Genome SNP Genotyping, and Linkage Analysis

Genomic DNA was extracted using the MagNA 96 DNA Isolation Kit (Roche Diagnostics, Mannheim, Germany). The concentration of the extracted DNA was measured using a NanoDrop spectrophotometer (Thermo Fisher Scientific, Waltham, MA, USA). DNA samples from the three affected individuals were subjected to WES, which was performed at the Medical Genetics Laboratories, Baylor College of Medicine (Houston, TX, USA). Exome capture and massively parallel sequencing were performed using VCRome 2.1 (Roche NimbleGen, Madison, WI, USA) and HiSeq (Illumina Inc., San Diego, CA, USA) with a 100 bp paired end mode.

After the possible causative variants were identified from WES, we performed whole genome single nucleotide polymorphism (SNP) genotyping to evaluate the segregation of the gene within the family members. Whole genome SNP genotyping was conducted using an Omni2.5 array chip (Illumina, San Diego, CA, USA), which contains ~2.4 million markers.

To confirm the *MBOAT7* genotype of the family members, polymerase chain reaction (PCR) and direct sequencing were performed using the BigDye Terminator Cycle Sequencing kit (Applied Biosystems, Foster City, CA, USA) on a 3100xl Genetic Analyzer (Applied Biosystems, Waltham, MA, USA). Primers for PCR and sequencing were designed using Primer3 (primers available on request) (11). Sequence assembly and variant detection were performed using Sequencher 5.0 (Gene Codes Corporation, Ann Arbor, MI, USA).

Linkage analysis was performed using SNP markers for the eight family members, including the three affected patients, three unaffected siblings, and their parents. The logarithm of odds (LOD) scores were calculated using MERLIN 1.1.2 with non-parametric linkage analysis (-npl) option (12).

We performed haplotype construction of the region harboring the c.757G>A variant of *MBOAT7* using PHASE v2.1.1 as stepwise expansion from 50 to 1,000 kb (13, 14).

### Molecular Modeling of MBOAT7

The amino acid sequence of human MBOAT7 was retrieved from the UniProt database (UniProt ID, Q96N66) (15). To provide a reliable model of human MBOAT7, several initial models were built using threading and various homology modeling approaches (Table 1 and Figure 1). After model building, the initial models were energy-minimized using 500 steps of steepest descent, followed by 250 steps of the conjugate gradient, using UCSF Chimera (16). Model quality was checked using PROCHECK (17) and protein structure analysis (ProSA) (18). A mutant model was generated by replacing Glu253 with Lys253 in the initial model, followed by the command-line implementation of the UCSF Chimera.

**Table 1:** Quality assessment of MBOAT7 models.

**Models**	**Online Web Server**	**Template**	**Most favored region (%)**	**Additional allowed region (%)**	**Generously allowed region (%)**	**Disallowed region (%)**	**G-Factor**	**ProSA Z-score**
Model-1	I-TASSER	6bugC	68.8	21.0	5.0	4.2	−1.01	2.39
Model-2	Swiss Model	6bugB	80.0	14.0	2.3	2.7	−0.31	−4.69
Model-3	Phyre2	6buhH	88.9	9.8	0.3	1.0	−0.32	−4.02
Model-4	M4T Server	^*^ –	84.7	13.2	1.7	1.6	−0.24	−5.29
Model-5	HHpred	6buhD	86.0	11.7	1.1	1.1	−0.25	−3.81

* *Template PDB ID and sequence identity have not yet been reported*.

### Molecular Dynamics Simulation

Molecular dynamics (MD) simulations of wild-type and mutant MBOAT7 models were carried out using the AMBER package (version 16) (19). All of the models were solvated in a rectangular box (8.0 Å) in the presence of counter ions to neutralize the system. Each system was individually minimized, heated, and equilibrated prior to MD simulations. We followed a two-step minimization procedure. In the first step, the protein model was fixed, whereas water and ions were minimized. In the second step, the restraint was removed, and the energy of the entire system was minimized. In these steps, 2,500 and 1,000 steps of the steepest descent, followed by 1,000 and 500 steps of the conjugate gradient, respectively, were applied. The systems were heated after minimization and equilibrated at 300 K for 100 ps using a constant volume. Once the systems were equilibrated, all restraints were removed under constant pressure before running further equilibration steps at 300 K. The same conditions of the final equilibration step were applied to the further production run of the MD simulations. The MD simulation trajectory files were saved every 0.5 ps, analyzed by the PTRAJ/CPPTRAJ module (20), and visualized in xmgrace (21).

### Trajectory Analysis

The simulated systems were monitored by analyzing each system using trajectory analysis. The CPPTRAJ module was used to analyze the root mean square deviation (RMSD) and root mean square fluctuation (RMSF). The RMSD values were calculated for both the wild-type and mutant structures, using the starting structure as a reference frame, and the deviation of the coordinates of a given set of atoms in a time interval. The RMSF was calculated to measure the fluctuations in each residue from their mean positions.

## Results

### Clinical Description of the Subjects

**Figure 2:**
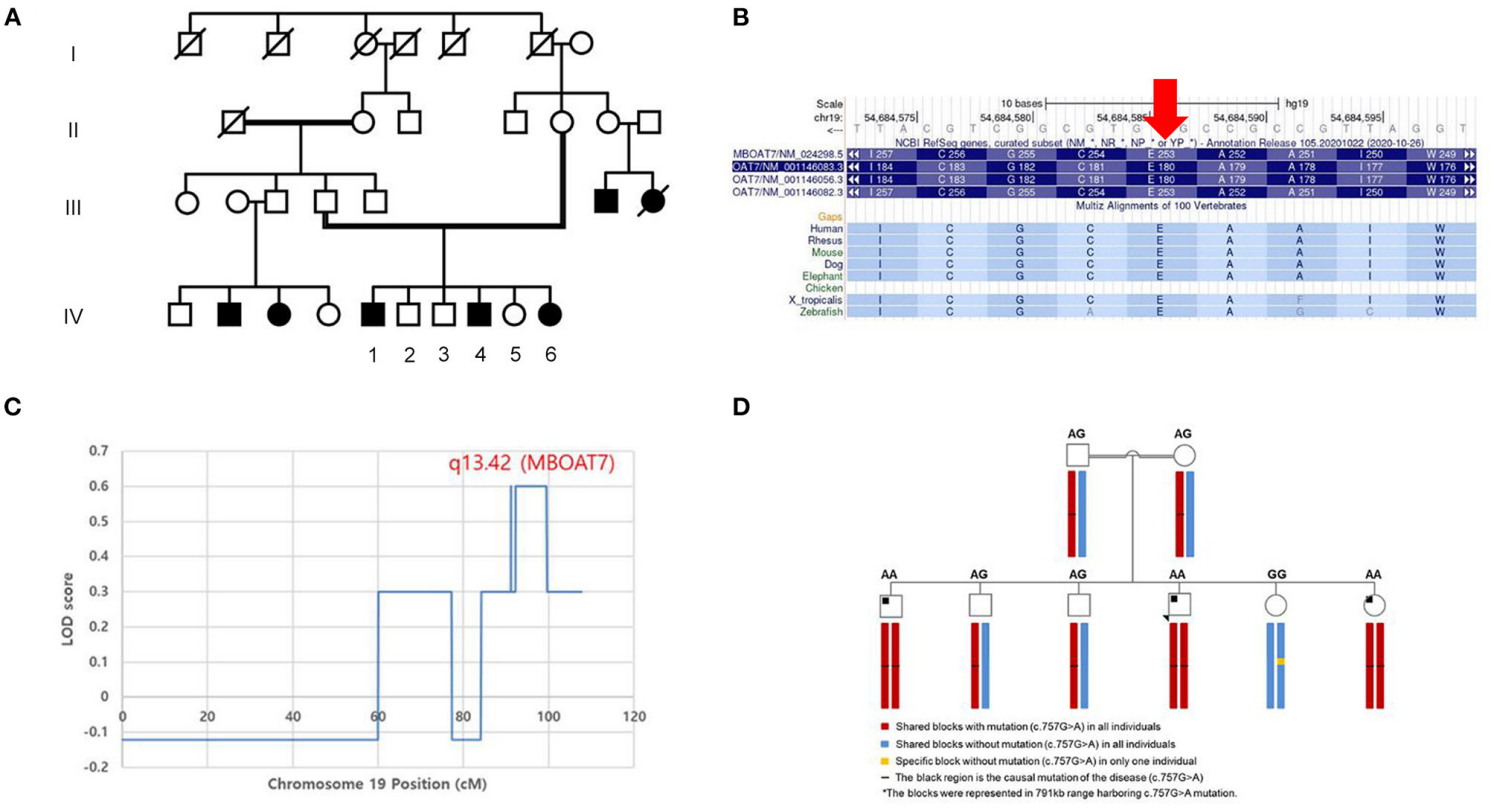
Pedigree and genetic analysis of the homozygous variant of *MBOAT7* (c.757G>A) in the consanguineous family. **(A)** The parents of the patients were distant relatives and there were four other family members (two paternal cousins and two maternal cousins) who had intellectual disabilities. Among the siblings, the three patients with a variant of *MBOAT7* had intellectual disability, motor developmental delay, and seizures in common. **(B)** The 253rd amino acid (glutamic acid), which is mutated in these three patients, is highly conserved across the species. The segregation analysis was conducted to determine the penetrance of the variant in this family. **(C)** The highest logarithm of the odds (LOD) score (which was twice that of the other regions) was found in the region of chromosome 19 (19q13.42), where *MBOAT7* is located (*P*-value < 0.05). **(D)** We performed haplotype analysis of the region harboring the c.757G>A variant of *MBOAT7*. A haploblock with the variant was shared by all of the individuals, except one unaffected wild-type sibling (IV-5). All affected individuals (IV-1, 4, and 6) were identified to have a homozygous haploblock, whereas their parents and two unaffected siblings (IV-2, 3) were heterozygous.

This consanguineous family included three affected individuals (two males and one female), three unaffected siblings, and their parents (Figure 2A). All affected individuals were born at full term with adequate birth weight, without any perinatal events. In infancy, they had a common history of hypotonia and global developmental delay (Table 2). There were four other family members (two paternal cousins and two maternal cousins) who had intellectual disabilities, of whom detailed information was not available.

**Table 2:** Clinical characteristics of three affected patients.

	**IV-1**	**IV-4**	**IV-6**
Sex/Age	M/22 y 11 months	M/13 y 8 months	F/4 y 10 months
Age at examination	19 y	9 y	2 y
Past medical history	Hypotonia in infancy	Hypotonia in infancy	Hypotonia in infancy
**Seizure**			
Onset age	5 y	6 months	3 months
Type	Generalized tonic-clonic Atypical absence	Focal impaired awareness Secondary generalized tonic	Generalized tonic Focal impaired awareness
Frequency	10–15/day at the beginning → 1–2/month at the time of visiting	1/month	Under control with medication
Antiseizure medications	Valproate, clonazapem, lacosamide, levetiracetam	Valproate	Levetiracetam
Delayed development	Severe global delay	Severe global delay	Moderate motor and adaptive delay Severe language delay
Psychosocial state	Severe intellectual disability Hyperactivity	Severe intellectual disability Hyperactivity	Severe cognitive dysfunction Behavioral problem is not proved yet.
Abnormal findings on neurologic examination	Slurred speech Clumsy hand movement Mild ataxic gait	Slurred speech Clumsy hand movement Mild ataxic gait	Hypotonia Mild ataxic gait
Disease course	Static	Static	Progressive → Static
EEG results	Sharp wave in Rt. or Lt. F region Ictal—rhythmic theta in both FC regions	Spike in Rt. or both F regions Spike in Rt. T region Polyspikes in Rt. hemisphere	Spike in Lt. or both FC regions
Brain MRI findings	Mild cerebral atrophy HSI in the bilateral globus pallidi and dentate nucleus	Mild cerebral atrophy HSI in the bilateral globus pallidi and dentate nucleus Perisylvian polymicrogyria	HSI in the bilateral globus pallidi and dentate nucleus

*F, frontal; FC, fronto-central; T, temporal; EEG, electroencephalography; MRI, magnetic resonance imaging; HSI, high signal intensity*.

Patient 1 (IV-1) was the first child of this family and was 19 years old at the first visit. He was born at full term and could walk independently after 2 years of age. He had intellectual disability and epilepsy. He did not have any dysmorphism, and his physical examination results were normal. His speech was slow, and his pronunciation was slurred. He had difficulty chewing and swallowing food. His hand movements were clumsy, and his gait was wide-based and mildly unsteady. Otherwise, his neurological examination results were within the reference limits. He had generalized tonic-clonic seizures that developed at the age of 5 years, with a frequency of up to 10 to 15 times per day. He was treated with three or more antiseizure medications; however, his seizures were not completely controlled. In addition, the patient showed prolonged atypical absence seizures. During his stay in our institution, his seizures decreased to one or two times per month while taking valproate, clonazepam, lacosamide, and levetiracetam. He had a severe intellectual disability, without an objective intelligence test, because he could not undergo a cognitive test. He could only communicate with his family members for basic daily conversations, using mostly single words and gestures. His speech was limited and difficult to understand, and he was unable to read or write. He could perform simple daily living tasks with the aid of other family members. EEG revealed frequent sharp wave discharges from the right, left, or midline frontal areas.

Patient 2 (IV-4) was 9 years old when he first visited our institution. He was born at full term without any perinatal problems. He was able to walk independently at the age of 2. Dysmorphism was not observed and the patient's physical examination results were normal. He was presumed to have a severe degree of intellectual disability, without a formal test for intelligence, because he could not make full sentences and could only say a few words. He could only understand a simple conversation. The patient was also unable to read or write. His hand movement was clumsy, and his gait was wide-based and unsteady; he could still walk independently. He could not hold pencils or spoons, because of his inept hand function. He could not stack blocks vertically, and he failed to perform the Purdue Pegboard Test. His pronunciation was slow and indistinct and he was unable to chew or swallow food. Fine and gross motor development were measured using the Bruininks-Oseretsky Test of Motor Proficiency, second edition (BOT-2). The standard scores of all four motor areas (fine manual coordination, manual coordination, body coordination, strength, and agility) were 20 (<1 percentile). Dysphagia with oral motor dysfunction was noted, especially when eating solid foods. Informal language tests revealed significant developmental delay in his language. The age equivalent of receptive language was 30 months, and the age equivalent of expressive language was 18 months. He had his first seizure at the age of 6 months, and the average frequency of his seizures was once a month with valproate treatment. EEG showed frequent sharp wave discharges from the right parietal, temporal, or both frontal areas.

Patient 3 (IV-6) was 24 months of age at the first visit. She was born at term, and her birth was uneventful. She could sit with assistance at the age of 8 months and walk alone at the age of 20 months. She mostly rode in a stroller because her gait was unstable and swayed from side to side. She began to show generalized seizures at the age of 3 months, and her EEG revealed frequent epileptiform discharges from the right or left fronto-central areas. She was started on levetiracetam, and her seizures were nearly under control. The Bayley Scales of Infant and Toddler Development were performed when she was 24 months old. Both the mental development index (score 84) and the psychomotor developmental indices (score 66) were <50 percentiles (her development age was 11 months). At the second visit, at 4 years of age, she showed a global developmental delay. The Peabody Developmental Motor Scale, 2nd edition (PDMS-2[D1]) was performed when she was 4 years old. The total motor quotient was 58 (gross motor quotient was 64, <1st percentile; fine motor quotient score, 61). Her capabilities and performance were very limited in all three domains of the Pediatric Evaluation of Disability Inventory: self-care, mobility, and social abilities; parental rating scales indicated that her developmental delay, in general, limited her adaptive functioning.

### Radiologic Findings

**Figure 3:**
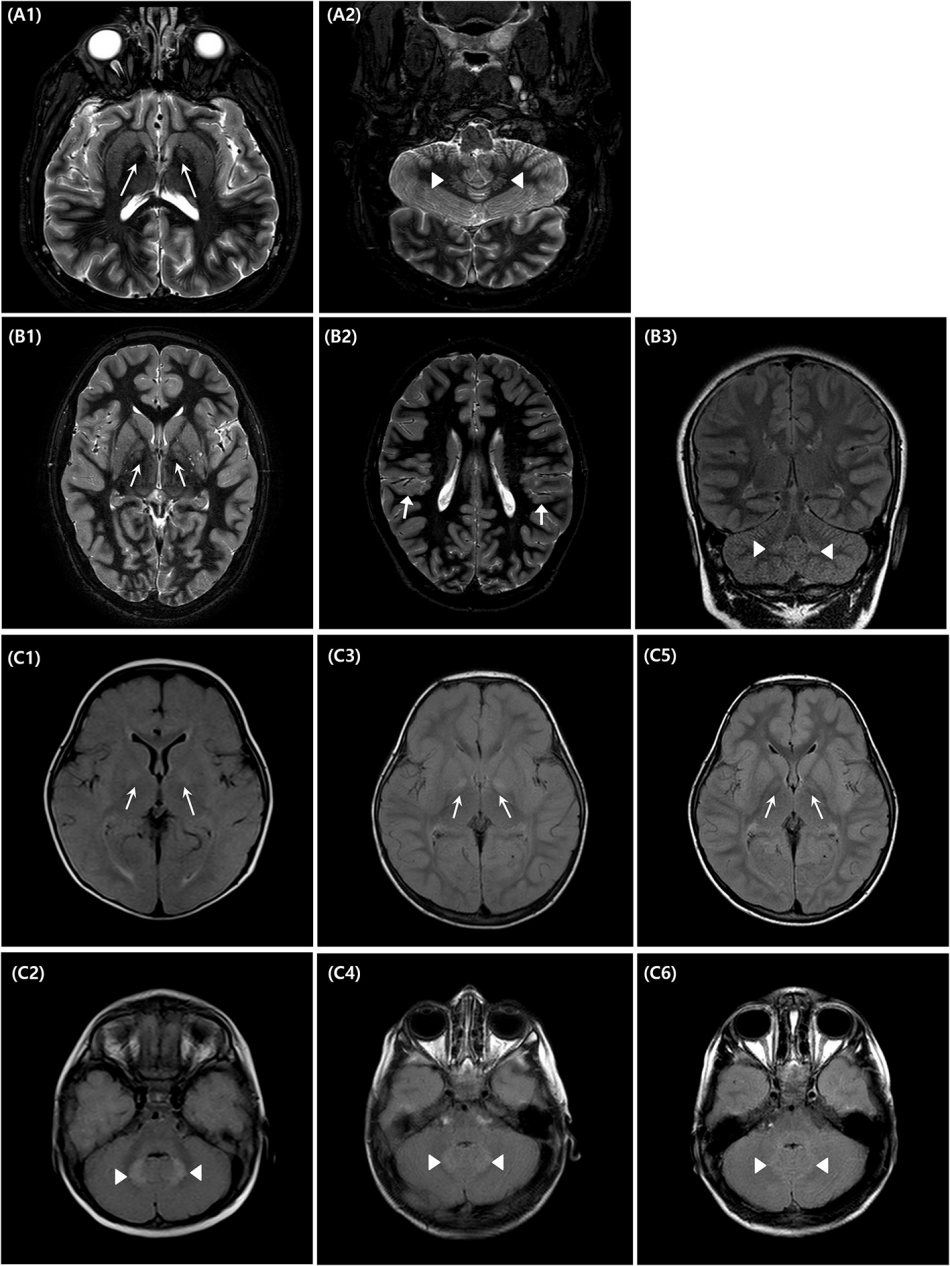
Brain magnetic resonance imaging of the affected individuals (IV-1, 4, 6). Three affected patients underwent brain magnetic resonance imaging (MRI) at different ages [**(A)**–Patient 1, **(B)**–Patient 2, **(C)**–Patient 3]. Patient 1 showed increased signal changes in the bilateral globus pallidi **(A1)** and the cerebellar dentate nuclei **(A2)** on T2-weighted axial images obtained at the ages of 22 years old. Patient 2 underwent brain MRI at the ages of 10 **(B1–B3)**. There are bilateral high signal intensity lesions in the same area as patient 1, on T2-weighted axial and fluid-attenuated inversion recovery coronal images, and additionally, bilateral polymicrogyria around the perisylvian area are seen [**(B2)**, arrow marks]. Patient 3 underwent a series of three brain MRIs when 8 months **(C1,C2)**, 2 years **(C3,C4)**, and 4 years old **(C5,C6)**, respectively. Brain MRI performed at 2 years of age, showed the most distinct high signal intensity of bilateral globus pallidi **(C3)** and cerebellar dentate nuclei **(C4)**.

Serial brain MRIs were performed at certain intervals in each patient: at the ages of 8 months, 2, and 4 years in patient 3, at the ages of 10 and 12 years in patient 2, and at the ages of 17 and 22 years in patient 1. All patients showed high signal intensity in the bilateral globus pallidi and cerebellar dentate nuclei on T2-weighted and fluid-attenuated inversion recovery images (Figure 3), which was the most prominent in patient 3. In patient 3, these findings were faint in the images at the age of 8 months and were the most distinct in the images at the age of 2 years (Figure 3C). In the last follow-up brain MRI of two patients (patient 2 and 3), the signal change in both areas became less prominent. In patient 1, these signal changes in follow-up MRI were more distinct than in the previous images. There was perisylvian polymicrogyria on the brain MRI of patient 2 (Figure 3B).

### Whole Exome Sequencing and *in-silico* Variant Effect Prediction

All three affected individuals had a homozygous NM_024298.4:c.757G>A (p.Glu253:Lys) variant in exon 6 of *MBOAT7* (chr19, GRCh37:g.54684587C>T), which was confirmed by direct sequencing. Direct sequencing revealed that both parents were heterozygous carriers. Unaffected siblings were carriers of either heterozygous (IV-2, 3) or wild-type (IV-5) alleles. The minor allele frequency was 0.0005% (1/201506 alleles) in the gnomAD database (v2.1.1). *In silico* prediction tools predicted the missense change as damaging (Align GVGD class C0; SIFT 0.00; PolyPhen-2 HumVar score 0.962). The 757th base is highly conserved across species (phyloP 5.45; phastCons 44 vertebrates 1.00), and the 253rd amino acid (glutamic acid) is highly conserved across species (Figure 2B).

### Whole Genome SNP Genotyping and Linkage Analysis

LOD scores > 2 were not found in this family. However, we found the highest LOD score (twice that of the other regions) in the region of chromosome 19 (19q13.42), where *MBOAT7* was located (*P*-value < 0.05) (Figure 2C). In haplotype analysis, all individuals showed the composition of three haploblocks (791 kb), depending on the presence of the c.757G>A variant (Figure 2D). A haploblock with the variant was shared by all individuals except for one unaffected wild-type sibling (IV-5). All affected individuals (IV-1, 4, and 6) were identified to be homozygous for the haploblock and their parents and two unaffected siblings (IV-2, 3) were heterozygous.

### Molecular Modeling of the Human MBOAT7

Human MBOAT7 was modeled using homology modeling and threading. We designed a computational pipeline (Figure 1) that can be used to build a reliable model, with low sequence similarity to known structures. In the first step, five initial models (model-1 to model-5, Table 1) were built using web-based modeling tools, by applying various modeling approaches. Notably, although templates in the protein data bank used for structure modeling in each method were different, they all belonged to the MBOAT family proteins (Table 1). Model-1 was built using the threading approach of I-TASSER, in which homologous fragments excised from the threading template, rather than a whole homologous template, were used for modeling (22, 23). The other four models (model-2 to model-5) were constructed using different homology modeling tools: the Swiss model (24), Phyre2 (25), M4T server (26), and HHpred (27). After energy minimization of each model, the quality of the initial models was evaluated using PROCHECK (17). For further validation, ProSA (18) was used to analyze the quality of the generated models. A model with a negative Z-score is considered to have a less erroneous structure (18). By considering residue geometry, G-factor, and ProSA Z-scores, model-3 was ultimately chosen as the most reliable model (Table 1). This model was used for further energy minimization, mutagenesis, MD simulation, and structure analysis (Figure 1). The sequence alignment of human MBOAT7 and a template is shown in Supplementary Material 2 with its secondary structure. In addition, ribbon diagrams of model-3 in the two orientations were compared with those of the template structure (Supplementary Material 3). The overall structural topology of human MBOAT7 was similar to that of the template, although the sequence identity was not high. The wild-type and mutant MBOAT7 models were used for MD simulation to investigate their structural stability and to compare them by monitoring RMSD and RMSF during 100 ns simulation time steps (Figure 1).

### Functional Interpretation of the Wild-Type and Mutant MBOAT7 Structures

**Figure 4:**
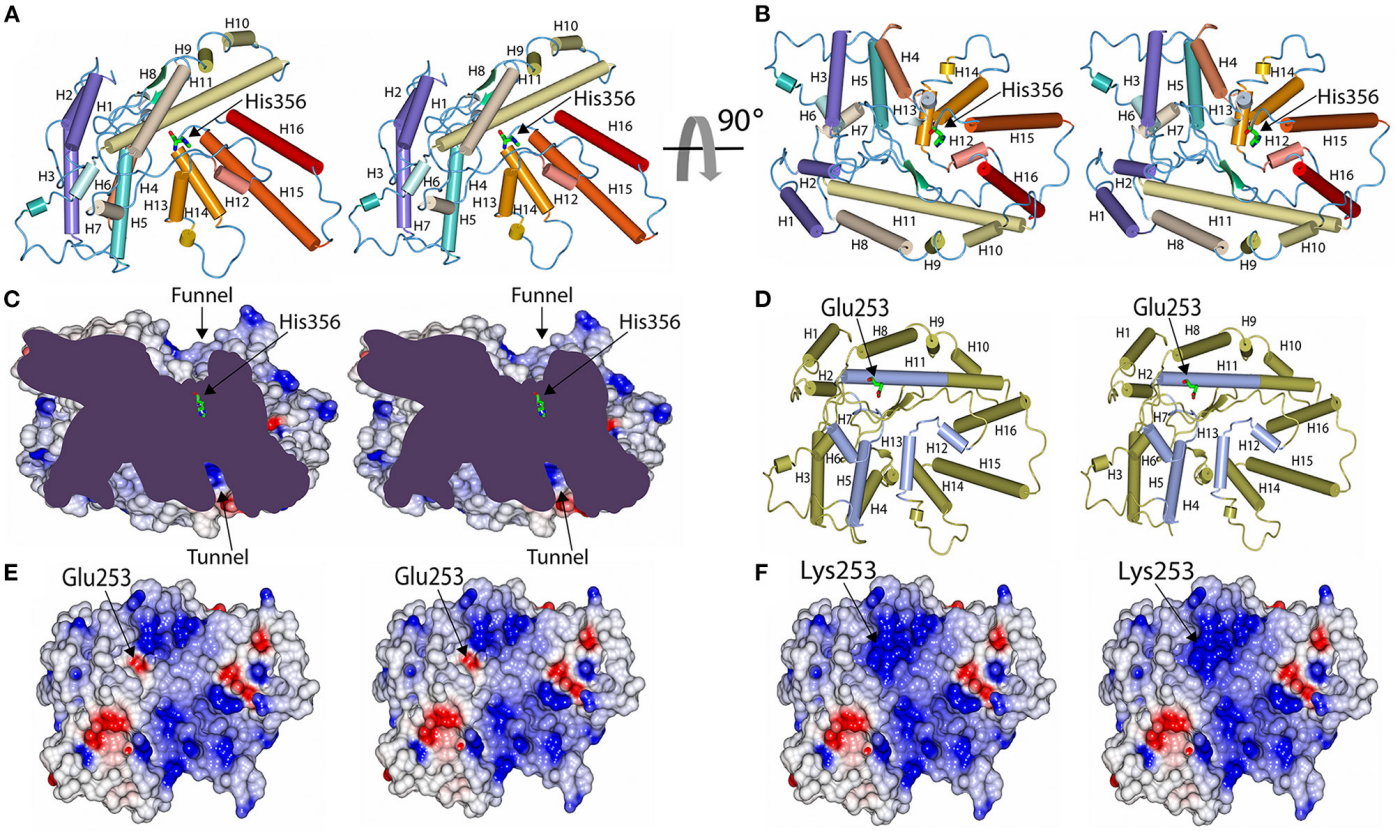
Model structure of human MBOAT7 protein and structural comparison between wild-type and variant MBOAT7. The human MBOAT7 model structures are drawn in two orientations with tube diagrams **(A,B)** and a cut-away surface charge distribution **(C)** in stereo views. The locations of the funnel which were oriented to the extracellular surface and the tunnel, which was directed to the cytosolic space, are indicated. The active site histidine residue (His356) was located at the bottom of the funnel. Tube diagram and surface charge distribution display in stereo views of the wild-type **(D,E)** and mutant MBOAT7 models **(F)** oriented to look down the funnel. In this orientation, the position of Glu253 in the wild-type **(E)** and Lys253 in the mutant **(F)** are shown in the middle of the funnel. Accordingly, the surface charge distribution of the funnel was altered by a mutation from Glu to Lys.

Human MBOAT7 has a 12% sequence identity with the bacterial homolog D-alanyl-lipoteichoic acid acyltransferase (DltB) (Supplementary Material 2), which also belongs to the MBOAT7 family of proteins. MBOAT family proteins commonly have a conserved helical core structure, comprising of an extracellular funnel and intracellular tunnel (28); this structure was also observed in MBOAT7 (Figures 4A–C). Both funnel and tunnel structures are important for the biochemical functions of the MBOAT family proteins, and they play a role in catalytic reactions (28). The catalytic site (His356), located at the bottom of the funnel, plays an important role in acyl transfer; thus, residues in the catalytic site are well-conserved in human MBOAT7 and in the template (Supplementary Material 2). For example, His336, found in the catalytic site of DltB, is well-aligned with His356 in human MBOAT7 (Supplementary Material 2). The funnel is formed with several residues from the transmembrane helices and connecting loops (Figures 4A–C); thus, it is expected that residues in the funnel are important for accommodating the substrates and are working as acyl acceptors. Glu253 was located on the surface of the funnel. Thus, the mutant MBOAT7 protein was expected to show an altered surface charge distribution (Figures 4D–F).

**Figure 5:**
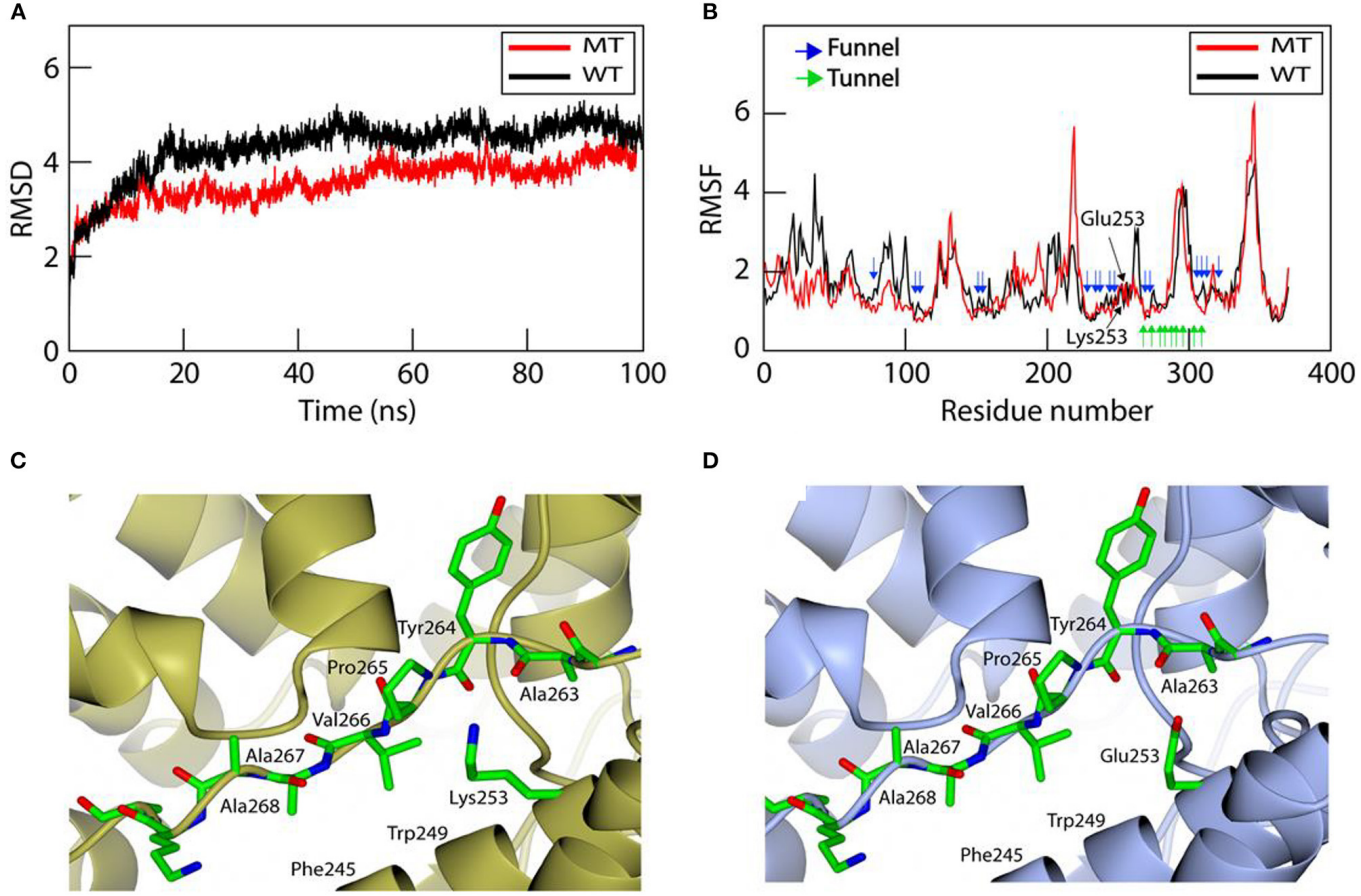
Molecular dynamics simulation for investigating the structural stability and structural comparison of the wild-type and mutant MBOAT7 models. **(A)** Root mean square deviation (RMSD) of the wild-type and mutant MBOAT7 models during 100 ns molecular dynamics simulation. The initial structure was used as the reference point for RMSD calculations. **(B)** The per residue root mean square fluctuation (RMSF) is calculated for the wild-type and mutant MBOAT7 models. The funnel, tunnel, and mutation site are indicated using blue, green, and black arrows, respectively. **(C)** Mutant and **(D)** wild-type MBOAT7 near the mutation site is shown using ribbon and stick models.

To further analyze the effect of mutation on the structural stability and on conformational changes, we examined the conformational flexibility of the overall structure of the wild-type and mutant MBOAT7 proteins by monitoring backbone RMSD values during MD simulations for 100 ns (Figure 5A). Both wild-type and mutant MBOAT7 models showed similar conformational behaviors for the initial 7 ns. However, after 7 ns, the wild-type protein showed higher conformational flexibility than the mutant protein, as the RMSD values of the wild-type protein were higher than those of the mutant protein (Figure 5A). The RMSF plots of the wild-type and mutant MBOAT7 models indicated that most residues located in the funnel of the mutant fluctuated less than those of the wild-type model (Figure 5B), suggesting that mutation at Glu253 may affect the structural stability of the funnel. Consistently, we observed that the variant amino acid, Lys253, is located close to the backbone carbonyl oxygens (Ala263 and Tyr264), in the loop located close to the helix (H11) containing Lys253 in the mutant protein (Figure 5C). The distances between the backbone carbonyl oxygens of Ala263 and Tyr264 and the helix containing Lys253 in the mutant protein are 3.0 Å and 3.5 Å, respectively, which are within the hydrogen bond range. However, the wild-type protein (Glu253) does not form such interactions due to the long distance among them (Figure 5D). This suggests that the hydrogen bonds in the mutant protein may contribute to the conformational rigidity of the funnel.

## Discussion

A prominent finding of this study is that molecular modeling of human MBOAT7 predicted the molecular pathomechanisms of the *MBOAT7* variant in patients with intellectual disabilities. This indicated that the variant amino acid (Lys253) is expected to affect funnel function and structural flexibility, resulting in MBOAT7 enzyme dysfunction. The variant amino acid is located on the funnel surface. It is expected to alter the surface charge distribution, which affects substrate specificity. In addition, the variant affected the structural flexibility of the funnel, which lowered the catalytic efficiency of the enzyme.

MBOAT7 plays an essential role in the incorporation of arachidonic acid into phosphatidylinositol, which is the main lipid component of mammalian membrane phospholipids (1, 29). Free cellular arachidonic acid is tightly regulated, and it is postulated that if arachidonic acid levels increase because of MBOAT7 deficiency, its pro-inflammatory metabolite might be harmful to cellular physiology (1, 30). The function of MBOAT7 was postulated using MBOAT7-null mice. The histological analysis of *Lpiat*^−/−^ mice showed a smaller cerebral cortex and hippocampus, abnormal cortical lamination, delayed neuronal migration, gyral abnormalities, and increased number of apoptotic cells in the cortex (10). They hypothesized that the change in the fatty acid composition of phosphatidylinositol phosphates may affect the interaction with its binding proteins and/or intramembrane localization of phosphatidylinositol phosphates in subcellular organelles. However, the mechanism by which this variant affects the function of the MBOAT7 protein remains unclear.

In this study, we identified a novel *MBOAT7* variant in siblings that shared clinical phenotype, intellectual disability, seizure, and common brain MRI findings. We modeled human MBOAT7 using homology modeling and threading to determine the pathophysiological mechanisms of this variant. In the protein modeling of MBOAT7, we predicted the functional role of the variant by identifying its location in the protein structure. MBOAT7 has a conserved helical core structure consisting of an extracellular funnel and intracellular tunnel, similar to other MBOAT family proteins (28). The variant amino acid located on the surface of the funnel was expected to alter the surface charge distribution, thus affecting substrate specificity.

MD simulations have been used to determine protein flexibility by predicting the time-dependent behavior of molecules (31). The conformational plasticity of the MBOAT7 funnel is key to determining functional changes in the enzyme. In MD simulations, the variant protein was expected to alter the conformational flexibility by changing the surface discharge contribution. With these structural changes in the variant protein, the function of MBOAT7, which transfers arachidonic acid from arachidonoyl-CoA to lysophosphatidylinositol and is essential for normal cortical lamination during brain development, is expected to decrease.

From the clinical point of view of the variant, the ages of the patients in the same family were diverse (2, 9, and 19 years of age), and they were followed up for 2 years. This was good for observing the natural course of patients with the *MBOAT7* variant. This disease seemed to progress until infancy with deterioration of motor function and more prominent lesions on brain MRIs. However, after the age, cognitive and motor dysfunction showed a static course that did not get worse. The developmental delay observed in the patients was similar to that reported in previous reports (1–7). All patients with *MBOAT7* variants showed infantile hypotonia and developmental delay in common. The patients in this study showed hypotonia in infancy; thereafter, they showed a marked delay in motor development. Language development was also significantly delayed and was even more prominent in expressive language functions. The representative symptoms in a previous report were intellectual disabilities and autism spectrum disorder (1). Of total 45 patients reported previously, 11 patients had autistic spectrum disorder and about one-thirds of them showed various behavioral problems such as aggression, hyperactivity, and self-injurious behavior (Supplementary Material 1). The patients in this study also had moderate-to-severe intellectual disabilities. They were hyperactive and inattentive, but their social interactions were much better than those of other functions, and their emotional expressions were relatively preserved. In addition, seizures were observed in most patients with *MBOAT7* variants, but the semiology and severity varied from patient to patient. Our patients also had epilepsy, but their epilepsy phenotypes were different. It was most problematic in patient 1, who had multiple seizure clusters in a day despite consuming three or more antiseizure medications. Although some authors reported patients having abnormalities in head circumference, there were no patients with macrocephaly or microcephaly in this study.

The patients in the present study showed prominent hand motor and oromotor dysfunctions. The two affected brothers had profound difficulty chewing and swallowing; the sister was bottle-fed until she was 2 years old. Their speeches were slow and slurred. Their gait was wide-based and staggering, which did not improve substantially despite rehabilitation treatment. This corresponded to ataxia and might be associated with lesions in the globus pallidus and dentate nucleus of the cerebellum found in these patients. Considering the functions of these regions, fine motor dysfunction, slurred speech, difficulty in chewing, and gait instability could be explained. In addition, one patient (patient 2) showed perisylvian polymicrogyria, similar to that reported previously. In that report, most patients carrying *MBOAT7* variants showed normal brain MRIs, except for two patients with brain atrophy and mild polymicrogyria (1).

Brain MRI of metabolic diseases can reveal specific findings according to the etiology. The involvement of the globus pallidus and cerebellar dentate nucleus may be related to problems in lipid metabolism. Zellweger's disease is one of the most severe lipid metabolism disorders. Brain imaging features include delayed myelination, perisylvian polymicrogyria, shortening of the bilateral globus pallidi on T1-weighted images, and hyperintense white matter in the hilus of the dentate nucleus on T2-weighted images (32–34). Neonatal adrenoleukodystrophy, another severe lipid metabolic disorder, shows diffuse brain atrophy and demyelination in the dentate nucleus and peridentate white matter (35). In contrast to patients with these severe lipid metabolic diseases, the signal changes in the globus pallidus and dentate nucleus in this study became less prominent as the patients grew older. This finding suggests that the pathological changes associated with an *MBOAT7* variant might occur in the early stages of brain development and might not have the effect of gradual substrate accumulation seen in other metabolic diseases. Another possible explanation is that the lesion appears more blurred owing to normal mineral deposition in the globus pallidus and dentate nucleus with aging.

In this study, molecular modeling of the variant protein revealed the molecular pathomechanism of a novel variant of *MBOAT7* (c.757G>A, p.Glu253Lys) in neurodevelopmental disorders. These results can be applied to other variants of this gene to determine its pathogenicity. To interpret sequence variants in patients, functional, population, computational and predictive, and segregation data are often required. Molecular modeling can be used to obtain reliable functional data to interpret the pathogenicity of newly detected genetic variants. Additionally, this study widened the clinical and radiological phenotypes and changes with the age of patients with *MBOAT7* variants. Further investigations using animal models to compare various variants through protein modeling will provide more evidence of the molecular pathomechanism of *MBOAT7*.

## Data Availability Statement

The original contributions presented in the study are publicly available. This data can be found here: https://www.ncbi.nlm.nih.gov/clinvar/, VCV001328055.1.

## Ethics Statement

The studies involving human participants were reviewed and approved by Institutional Review Board of Samsung Seoul Hospital (IRB No. 202007-132-004). Written informed consent was obtained from all participants and/or the participants' legal guardian to participate in this study and for the publication of any potentially identifiable images or data included in this article.

## Author Contributions

JiL clinically characterized the patients, analyzed the data, and wrote the manuscript. AS and KKK performed molecular modeling and prepared the manuscript. JP and JHJ did the genetic analysis. JHK analyzed the brain images. JYK executed functional evaluation of the patients. JWK and JeL designed the study and supervised the study progress. All authors read and approved the final manuscript.

## Funding

This study was supported by the Future Medicine 2030 Project of the Samsung Medical Center [SMX1210841].

## Conflict of Interest

The authors declare that the research was conducted in the absence of any commercial or financial relationships that could be construed as a potential conflict of interest.

## Publisher's Note

All claims expressed in this article are solely those of the authors and do not necessarily represent those of their affiliated organizations, or those of the publisher, the editors and the reviewers. Any product that may be evaluated in this article, or claim that may be made by its manufacturer, is not guaranteed or endorsed by the publisher.

## Supplementary Material

Supplementary Material 1 *MBOAT7* variants reported previously in neurodevelopmental disorders.

Supplementary Material 2 Sequence alignment of human MBOAT7 (query sequence) with D-alanyl-lipoteichoic acid acyltransferase (DltB; template sequence).

Supplementary Material 3 Structures of D-alanyl-lipoteichoic acid acyltransferase and MBOAT7.
