# Supplementary material for: Functional and Structural Changes in the Membrane-Bound O-Acyltransferase Family Member 7 (MBOAT7) Protein: The Pathomechanism of a Novel MBOAT7 Variant in Patients With Intellectual Disability
Source: Front Neurol. 2022 Apr 18;13:836954. doi: 10.3389/fneur.2022.836954 (PMC9058081; doi:10.3389/fneur.2022.836954)
Supplement: Supplementary Material 1 — MBOAT7 variants reported previously in neurodevelopmental disorders. [file Table_1.DOCX]

**Supplementary Table 1. MBOAT7 variants reported to date associated with neurodevelopmental disorders**

| **References** | **Transcript** | **c.DNA position** | **Amino acid change** | **Number of Individuals / Families** | **Phenotypes (number of affected patients)** | **MRI findings** |
| --- | --- | --- | --- | --- | --- | --- |
| Johansen et al.(1) | NM_024298.3 | c.758_778del | p.(Glu253_Ala259del) | 5 / 2 | DD, seizure (4), microcephaly (1), autism (4) | Polymicrogyria, cortical atrophy |
|  | NM_024298.3 | c.820_826del | p.(Gly274Prfs*47) | 4 / 1 | DD, seizure (1) | Not known |
|  | NM_024298.3 | c.126_145del | p.(Leu43Hisfs*8) | 3 / 1 | DD, seizure (3) | Unremarkable |
|  | NM_024298.3 | c.423delG | p.(Leu142Cysfs*8) | 2 / 1 | DD, seizure (1) | Not known |
|  | NM_024298.3 | c.854+1G>C | Not applicable | 2 / 1 | DD, seizure (1) | Not known |
| Hu et al.(2) | NM_024298.3 | c.1069G>A | p.(Gly357Ser) | 3 / 1 | Intellectual disability, seizure, aggression (3), hyperactivity (1), self-injurious behavior (2) | leukoencephalopathy |
| Santos-Cortez et al.(3) | NM_024298.3 | c.251delT | p.(Leu84Argfs*25) | 4 / 1 | DD, microcephaly (2) | Not known |
| Yalnizoglu et al.(4) | NM_024298.3 | c.1278G>A | p.(Trp426*) | 3 / 1 | DD, seizure (2) | Cerebellar dysgenesis and atrophy, polymicrogyria, bilateral globus pallidus signal change, dentate nuclei hyperintensity, thin corpus callosum, enlarged perivascular spaces |
|  | NM_024298.3 | c.259C>T | p.(Arg87Gln) | 2 / 1 | DD, seizure, autism (1) |  |
|  | NM_024298.3 | c.680_690del | p.(Leu227Profs*65) | 2 / 2 | DD, seizure, autism (2) |  |
|  | NM_024298.3 | c.1126G>A | p.(Glu376Lys) | 1 / 1 | DD, seizure, autism (1) |  |
| Jacher et al.(5) | NM_024298.3 | c.558-2A>G | Not applicable | 1 / 1 | DD, macrocephaly, seizure, autism, hyperactivity | leukoencephalopathy |
| Khan et al.(6) | NM_024298.3 | c.758_778del | p.(Glu253_Ala259del) | 7 / 2 | DD, microcephaly (2), seizure (6), aggression (3), hyperactivity (1) | Cortical atrophy |
| Farne et al.(7) | NM_024298.4 | c.1057_1058delGCinsCA | p.(Ala353His) | 1 / 1 | DD, seizure, macrocephaly, hyperactivity, aggression | Cerebellar dysgenesis |
| Heidari et al.(8) | NM_024298.5 | c.1062C>A | p.(Tyr354*) | 2 / 1 | DD, seizure, autism (2) | Bilateral globus pallidus signal change, thin corpus callosum (50%), brain atrophy (50%) |
|  | NM_024298.5 | c.1135del | p.(Leu379Trpfs*9) | 1 / 1 | DD, seizure, hyperactivity | Globus pallidus signal change, dentate nuclei hyperintensity |
| Sun et al.(9) | NM_024298.3 | c.758_778del | p.(Glu253_Ala259del) | 6 / 2 | DD, seizure (3), feeding refusal (3), self-injurious behavior (4) | Not known |

**Abbreviations**

DD, developmental delay

**References**

1. Johansen A, Rosti RO, Musaev D, Sticca E, Harripaul R, Zaki M, et al. Mutations in MBOAT7, Encoding Lysophosphatidylinositol Acyltransferase I, Lead to Intellectual Disability Accompanied by Epilepsy and Autistic Features. Am J Hum Genet. 2016;99(4):912-6.

2. Hu H, Kahrizi K, Musante L, Fattahi Z, Herwig R, Hosseini M, et al. Genetics of intellectual disability in consanguineous families. Mol Psychiatry. 2019;24(7):1027-39.

3. Santos-Cortez RLP, Khan V, Khan FS, Mughal ZU, Chakchouk I, Lee K, et al. Novel candidate genes and variants underlying autosomal recessive neurodevelopmental disorders with intellectual disability. Hum Genet. 2018;137(9):735-52.

4. Yalnizoglu D, Ozgul RK, Oguz KK, Ozer B, Yucel-Yilmaz D, Gurbuz B, et al. Expanding the phenotype of phospholipid remodelling disease due to MBOAT7 gene defect. J Inherit Metab Dis. 2019;42(2):381-8.

5. Jacher JE, Roy N, Ghaziuddin M, Innis JW. Expanding the phenotypic spectrum of MBOAT7-related intellectual disability. Am J Med Genet B Neuropsychiatr Genet. 2019;180(7):483-7.

6. Khan S, Rawlins LE, Harlalka GV, Umair M, Ullah A, Shahzad S, et al. Homozygous variants in the HEXB and MBOAT7 genes underlie neurological diseases in consanguineous families. BMC Med Genet. 2019;20(1):199.

7. Farne M, Tedesco GM, Bedetti C, Mencarelli A, Rogaia D, Colavito D, et al. A patient with novel MBOAT7 variant: The cerebellar atrophy is progressive and displays a peculiar neurometabolic profile. Am J Med Genet A. 2020;182(10):2377-83.

8. Heidari E, Caddeo A, Zarabadi K, Masoudi M, Tavasoli AR, Romeo S, et al. Identification of novel loss of function variants in MBOAT7 resulting in intellectual disability. Genomics. 2020;112(6):4072-7.

9. Sun L, Khan A, Zhang H, Han S, Habulieti X, Wang R, et al. Phenotypic Characterization of Intellectual Disability Caused by MBOAT7 Mutation in Two Consanguineous Pakistani Families. Front Pediatr. 2020;8:585053.
