# Supplementary figures and images for: Functional and Structural Changes in the Membrane-Bound O-Acyltransferase Family Member 7 (MBOAT7) Protein: The Pathomechanism of a Novel MBOAT7 Variant in Patients With Intellectual Disability
Source: Front Neurol. 2022 Apr 18;13:836954. doi: 10.3389/fneur.2022.836954 (PMC9058081; doi:10.3389/fneur.2022.836954)

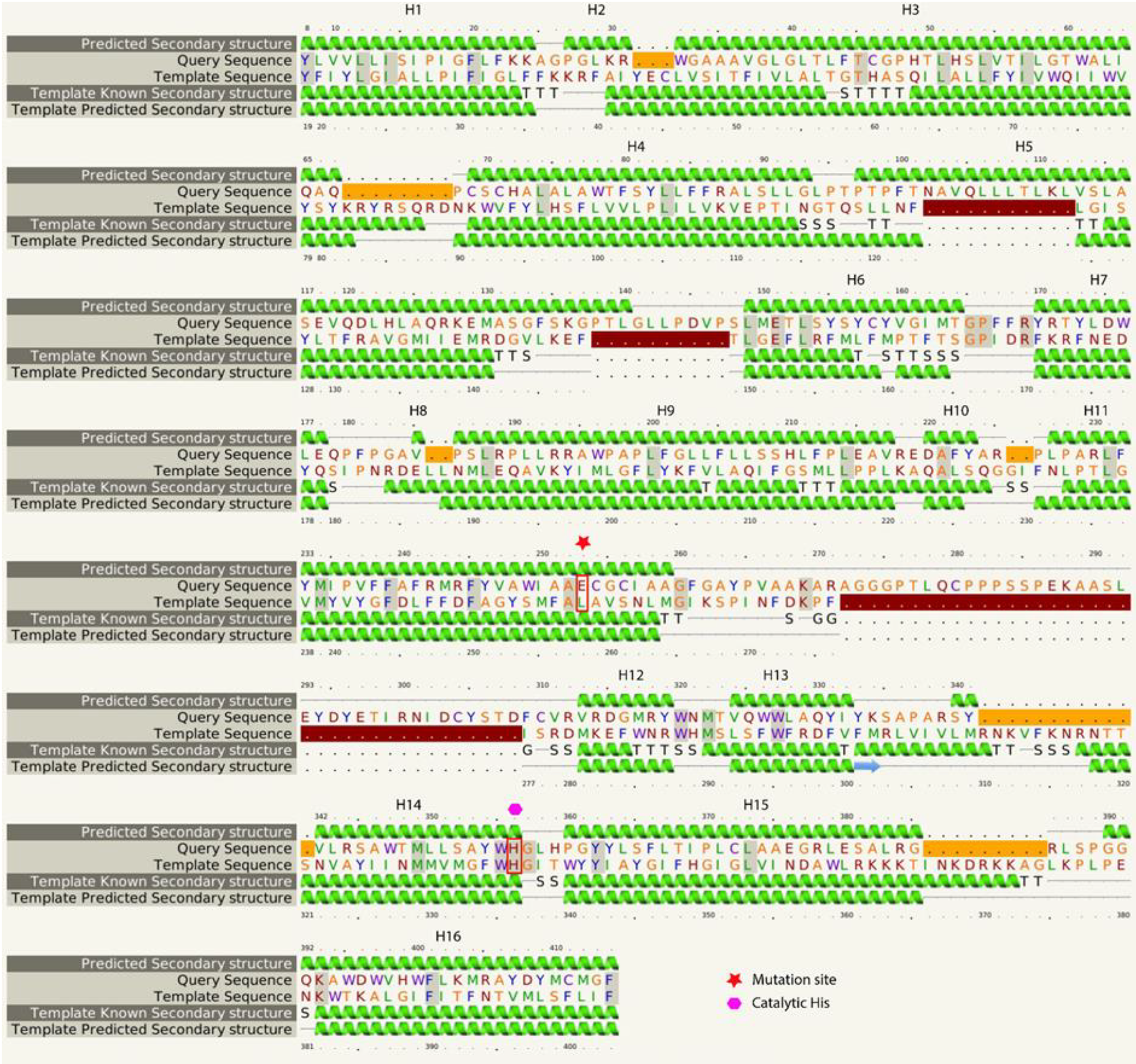

Supplement: Supplementary Material 2 — Sequence alignment of human MBOAT7 (query sequence) with D-alanyl-lipoteichoic acid acyltransferase (DltB; template sequence). [file Image_1.TIF]

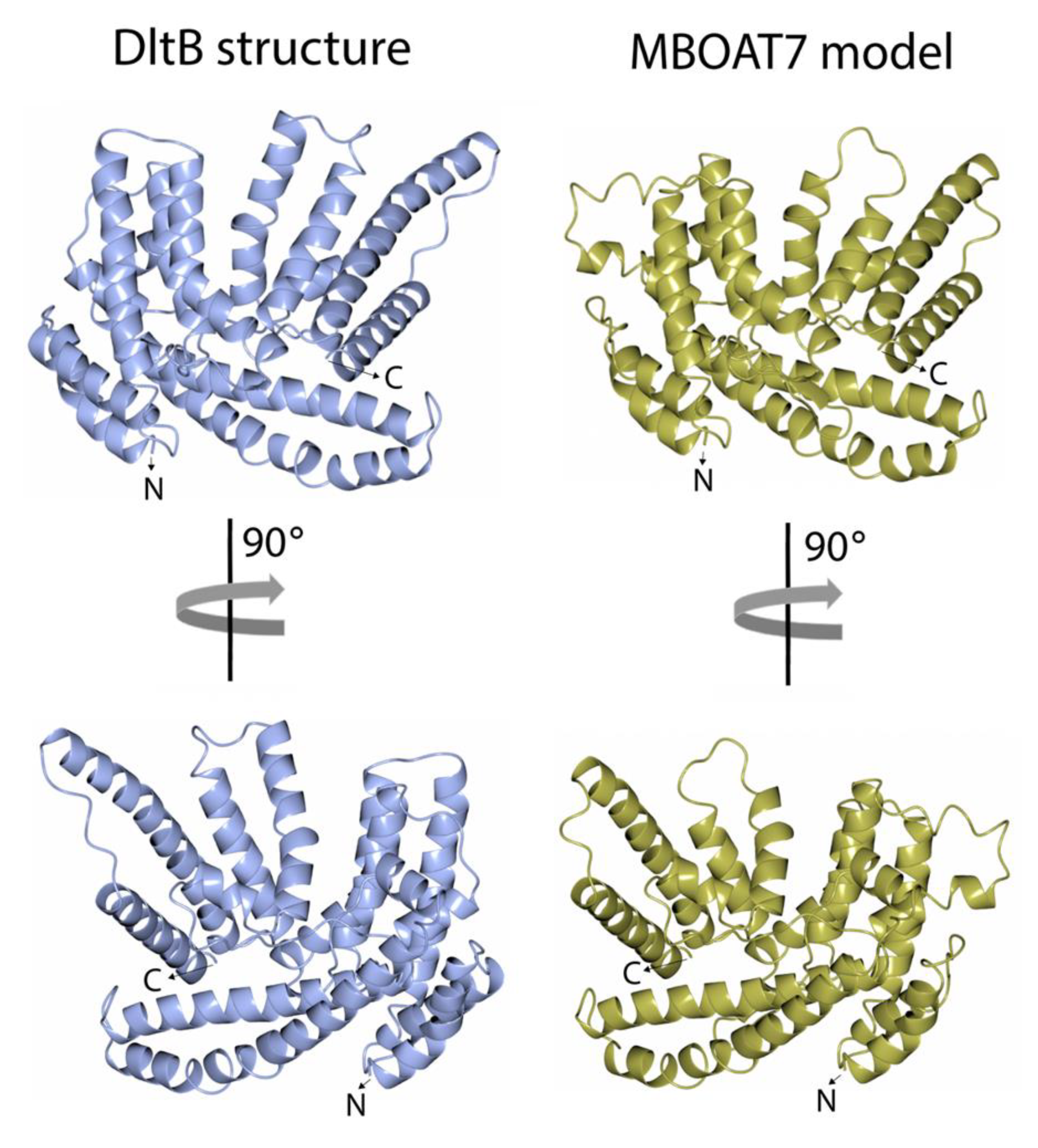

Supplement: Supplementary Material 3 — Structures of D-alanyl-lipoteichoic acid acyltransferase and MBOAT7. [file Image_2.TIF]
